# Safety and efficacy of switching to elvitegravir, cobicistat, emtricitabine, tenofovir disoproxil fumarate in treatment-experienced people with HIV: a multicenter cohort study

**DOI:** 10.1186/s12981-022-00499-4

**Published:** 2023-01-03

**Authors:** Nathalie De Castro, Alexandre Brun, Pierre Sellier, Gwenn Hamet, Frédéric Mechaï, Valérie Garrait, Amélie Chabrol, Marie-Anne Bouldouyre, Eric Froguel, Didier Troisvallets, Pauline Caraux-Paz, Constance Delaugerre, Willy Rozenbaum, Jean-Michel Molina

**Affiliations:** 1 Infectious Diseases Department, APHP-Hôpital Saint-Louis, 1 Avenue Claude Vellefaux, 75010 Paris, France; 2 COREVIH Ile-de-France Est, Paris, France; 3 APHP-Hôpital Lariboisière, Paris, France; 4 APHP-Hôpital Avicenne, Bobigny, France; 5 Centre Hospitalier Intercommunal de Créteil, Créteil, France; 6 Centre Hospitalier Sud-Francilien, Corbeil-Essonnes, France; 7 Centre Hospitalier Intercommunal Robert Ballanger, Aulnay-Sous-Bois, France; 8 Grand Hôpital de l’Est Francilien, Jossigny, France; 9 Centre Hospitalier de Gonesse, Gonesse, France; 10 Centre Hospitalier de Villeneuve St. Georges, Villeneuve Saint-Georges, France; 11 Université de Paris, Paris, France

**Keywords:** E/C/F/TDF, switch, virologic efficacy, integrase resistance

## Abstract

**Objectives:**

We assessed the virologic efficacy of switching to co-formulated elvitegravir, cobicistat, emtricitabine, tenofovir disoproxil fumarate (E/C/F/TDF) in patients with controlled HIV infection.

**Methods:**

We conducted a retrospective multicenter observational cohort study including adult patients with controlled HIV-1 infection on any stable antiretroviral (ART) regimen, who switched to E/C/F/TDF. Success was measured by the proportion of patients with plasma viral load < 50 copies/ml at W48 using the FDA snapshot algorithm. We also assessed risk factors associated with virological failure (VF).

**Results:**

382 patients with HIV RNA < 50 copies/mL who switched to E/C/F/TDF were included in the study. Most patients (69.9%) were male, with median age 44 years (IQR 38–51), who had been on ART for a median of 7 years (IQR 4–13). Median CD4 count was 614/mm^3^ and 24.6% of the patients had a history of previous virological failure. The reasons for switching were simplification (67.0%) and tolerance issues (22.0%). At week 48, 314 (82.0% [95% CI 78.4–86.0]) patients had HIV RNA < 50 copies/mL, 13 (3.5% [95% CI 3.64–8.41]) experienced virological failure. Genotype at failure was available in 6/13 patients with detection of resistance-associated mutations to integrase inhibitors and NRTIs in 5/6 (83.3%) patients. We found no predictive factor associated with virological failure except for a borderline significance with the duration of viral suppression before the switch. Tolerability of E/C/F/TDF was good with 23/382 (6.0%) patients experiencing mild adverse reactions.

**Conclusion:**

In our cohort, switching well-suppressed patients to E/C/F/TDF resulted in few virologic failures and was well tolerated. However, resistance to integrase inhibitors emerged in patients with virological failure.

## Introduction

The availability of single tablet regimens (STR) started with the release of Atripla® (efavirenz (EFV)/emtricitabine (FTC)/tenofovir disoproxil fumarate (TDF)) and has improved the effectiveness of antiretroviral therapy (ART), with more convenient regimens as a result of reduced pill burden [1]. Integrase inhibitors (INSTIs) are now recommended as first-line regimens and are available as STRs in Europe [2–14]. Co-formulated elvitegravir, cobicistat, emtricitabine and tenofovir disoproxil fumarate or tenofovir alafenamide (E/C/F/TDF and E/C/F/TAF) have been approved for the treatment of naïve people with HIV (PWH), with very high virologic efficacy and have also been evaluated in switch studies in patients with controlled HIV-1 infection [8, 9, 11, 12, 15, 16]. Two open-label phase 3 trials demonstrated that in PWH patients virologically suppressed on protease inhibitors (PI) or non-nucleoside reverse transcriptase inhibitor (NNRTI)-based regimens, switching to E/C/F/TDF was non-inferior at W48 and W96 to maintaining their background regimen [8, 9, 15, 16]. Patients had no prior documented resistance to emtricitabine and/or TDF and the rate of virological failure after the switch was very low [8, 9, 15, 16]. In terms of tolerance, there was no difference in renal events leading to drug discontinuation between the switch group and the PI and NNRTI groups [8, 9, 15, 16]. In STRATEGY NNRTI, there was a significant reduction in the prevalence of nightmares and intense dreams, dizziness and fatigue/loss of energy in patients switching from efavirenz [8].

E/C/F/TDF was the second STR approved in France in February 2014 for the treatment of HIV infection in naïve patients. After that date, clinicians switched patients to E/C/F/TDF for various reasons, including convenience, tolerance, or patient’s request. In routine clinical care, the patients’ characteristics differ from those selected to participate in the STRATEGY studies, which could impact virologic response.

We wished to assess the efficacy and safety of switching to E/C/F/TDF in a standard clinical setting. We hypothesized that non-selected, PWH, well suppressed on NNRTI, boosted PI or INSTI-based ART, should maintain virologic suppression at W48 after switching to E/C/F/TDF, without major tolerability issues.

## Methods

COREVIH IDF Est is a group of 27 clinical centers located in the Paris area, with approximately 11,000 HIV-infected patients on follow-up in 2014. All centers use the computerized Nadis® database as an electronic health record (EHR) for patient follow-up and care. Demographic data, clinical outcomes, ART history and laboratory tests (CD4 T cell count and HIV-1 RNA) are collected at each patient’s visit by the clinician, the rate of completion is monitored and is > 90%. In the EHR, the reason for any ART change has to be reported to be able to print the patient’s prescription, so the reason for the switch is accurately reported.

### Patients

We conducted a retrospective multicenter observational cohort study within the COREVIH IDF Est clinical centers enrolling ART-experienced patients. Eligible patients were adult patients (≥ 18 years old) with controlled HIV-1 infection on any stable ART regimen (no limit on the previous number of regimens) who switched to E/C/F/TDF between February 2014 (date of approval of E/C/F/TDF in France) and August 2015. Controlled HIV-1 infection was defined by plasma HIV-1 RNA < 50 copies/mL without ART modification in the previous 12 months.

Before enrollment in the Nadis® database, it is required that patients sign an informed consent form which was approved by the Paris Saint-Louis ethics committee and Commission Nationale Informatique et Libertés (CNIL) allowing the use of patients’ data for non-interventional studies without the need for further consent in accordance with French regulations (CNIL, number 1,171,457).

### Study procedures

Our primary objective was to assess the efficacy and tolerance of the switch to E/C/F/TDF measured by determination at W48 of the proportion of patients still on E/C/F/TDF and with plasma HIV-1 RNA < 50 copies/mL.

At baseline (date of the switch), the following data were extracted: date of HIV infection, date of first ART, number of previous ART and type of regimen, nadir CD4 T cell count or CD4 T cell count at ART initiation, CD4 T cell count (within 6 months before the switch), the reason for switching to E/C/F/TDF, creatinine levels and estimated Glomerular filtration rate (eGFR) using MDRD, history of prior virologic failure and previous genotypes. If the genotypes were not available in the database for patients who failed (at baseline and at virological failure), we went back to the paper chart or contacted the virology laboratory to collect the information. During the study period, genotypes were performed using Sanger sequencing. In France, it is usually recommended to perform genotyping in case of confirmed virologic failure with HIV RNA ≥ 200 copies/mL.

During the 48 weeks of follow-up, any reason for ART change was collected, any plasma HIV-1 RNA measure between baseline and W48, clinical adverse events reported at each visit and creatinine levels at W48 were also collected.

### Study endpoints

Our primary endpoint was the proportion of success defined as plasma HIV-1 RNA < 50 copies/mL using the FDA snapshot algorithm [17]. Due to the retrospective design of this real-life study, we defined a window period for the primary endpoint analysis of − 12 weeks/+ 24 weeks to limit missing values.

Virologic success was thus defined as plasma HIV-1 RNA below 50 copies/mL at week 48 with a window period of W36 to 72 weeks and no discontinuation of E/C/F/TDF.

The secondary endpoints were: reasons for the switch as reported in the patient’s EHR (simplification, patient demand, tolerance, other or unknown); the proportion of patients with virological failure defined as a confirmed viral load ≥ 50 copies/ml at two consecutive measurements within one year after the switch or one HIV RNA ≥ 50 copies and E/C/F/TDF discontinuation; resistant mutations in case of virologic failure; reasons for treatment discontinuation after the switch and tolerability of the treatment; risk factors for virologic failure.

Of note, INSTI resistance testing in ART-naïve patients before ART initiation was not standard of care in France before 2014 but was performed in case of virological failure since 2008.

### Statistical analysis

The primary analysis was an intent-to-treat (ITT) analysis using the FDA snapshot algorithm at W48. Patients were classified in the following categories: (i) virological success defined as plasma viral load < 50 copies/ml at W48 (time window W36-W72) while on E/C/F/TDF; (ii) virologic failure: plasma viral load ≥ 50 copies/ml at any visit after the switch; (iii) no data: missing data or lost to follow-up and discontinuation of E/C/F/TDF for reasons other than virologic failure at a prior visit.

Data are presented as median with IQR for continuous variables and frequencies with percentages for qualitative variables. Categorical variables were compared using Fisher’s exact tests while quantitative variables were compared using Wilcoxon signed-rank tests.

Comparisons between patients with virological failure and virological success were performed to assess if the following baseline risks factors were associated with virological failure: age, gender, mode of HIV infection acquisition, use of INSTI or PI/r before switch, time since HIV infection diagnosis, duration of ART, duration of viral suppression before the switch, nadir and baseline CD4 T cell counts, creatinine plasma level, previous resistance-associated mutations (RAM) to at least one drug of the regimen.

All tests were two-sided at the 0.05 significance level. Analyses were performed using R statistical package (version 3.2.2: the R Foundation, Vienna, Austria).

## Results

Among the 10,128 patients on ART during the study period, 661 were receiving E/C/F/TDF and 382 were included in the present study. The two main reasons for non-inclusion were patients receiving E/C/F/TDF as initial ART regimen (n = 137) and HIV RNA ≥ 50 copies before the switch (n = 114). The study flow chart is reported in Fig. 1. Baseline characteristics of the patients are reported in Table 1.

**Table 1:** Patients characteristics at baseline (n = 382)

	Present study n = 382	Strategy–PI Arribas et al. [10] n = 293	Strategy– NNRTI Pozniak et al. [9] n = 291
Male gender	267 (69.9)	250 (85.0)	268 (92.0)
Age (Years)	44 [38 − 5]	41 [33–48]	43[34–49]
Region of origin		NA	NA
Europe	181 (48.0)		
Sub–Saharan Africa	135 (35.8)		
North Africa	22 (5.8)		
Other	39 (10.3)		
Mode of HIV infection		NA	NA
Heterosexual	189 (49.5)		
MSM	151 (39.5)		
Intravenous drug users, transfusion, others	42 (11.0)		
Time since HIV infection diagnosis (years)	11 [5–16]	4 [3–7]	5 [3–7]
History of ART change because of virologic failure	95 (24.6)	0	0
Nadir CD4 cell count (cell/mm^3^)	232 [108–353]		
CD4 cell count at baseline (cell/mm^3^)	614 [450.7–812.7]	564 [423–757]	561 [450–722]
Creatinine levels at baseline (µmoL/L)	79 [70–89]		
eGFR (mL/min)	NA	111.2 [96.0–127.9)	114.4 [99.7–132.4]
Time between first ART and switch to E/C/F/TDF (years)	7 [4–13]	3 [2–4]	4 [2–5]
Duration of HIV RNA < 50 copies/mL before the switch, months	32 [11–62]		
On first or second ART regimen before the switch	95 (25.0)	293 (100.0)	291 (100.0)
ART before the switch			
2NRTI + 1PI/r^§^	195 (51.0)	293 (100.0)	0
2NRTI + 1NNRTI^§§^	84 (22.0)	0	291 (100.0)
2 NRTI + 1INSTI^§§§^	67 (17.5)	0	0
Other PI/r–based regimens	16 (4.2)	0	0
Other combinations	20 (5.2)	0	0
Reason for the switch		NA	NA
Simplification	256 (67.0)		
Tolerance	85 (22.3)		
Patients demand	4 (1.1)		
Other or unknown*	37 (9.7)		

Data are n (%) or median [IQR]

*MSM* men who have sex with men, *ART* antiretroviral therapy, *eGFR* estimated glomerular filtration rate, *NRTI* nucleoside reverse transcriptase inhibitors, *NNRTI* non-nucleoside reverse transcriptase inhibitors, *PI/r* protease inhibitor boosted with ritonavir, *INSTI* integrase strand transfer inhibitor. NA data not available

^§^TDF/FTC + DRV/R n = 80, ABC/3TC + DRV/r n = 9, TDF/FTC + ATV/R n = 70, ABC/3TC + ATV/r n = 11, TDF/FTC + LPV/R n = 14, ABC/3TC + LPV/r n = 1, other 2NRTI + PI/r n = 11

^§§^TDF/FTC/EFV n = 43, TDF/FTC/RPV n = 28, other NNRTI containing regimens n = 13

^§§§^TDF/FTC + RAL n = 59, ABC/3TC + RAL n = 2, ABC + TDF + RAL n = 1, TDF/FTC + DTG n = 2 ABC/3TC + DTG n = 2, ABC/3TC/DTG n = 1

*Prevention of toxicity (n = 15), end of protocol (n = 7), drug-drug interaction (n = 4), adherence issues (n = 3), treatment failure (n = 3), unknown reason (n = 5)

Patients were mostly male (69.9%), with median age of 44 years, 42.0% were born in Africa and 89.0% had been infected via sexual transmission (59% heterosexual and 40% men who have sex with men). At the time of the switch to E/C/F/TDF, the median duration of HIV infection was 11 years (IQR: 5–16) and the median time from ART initiation was 7 years (IQR 4–13). Before switching to E/C/F/TDF, a majority of patients received a combination of two NRTI and a third agent: ritonavir- boosted PI (PI/r) in 195/382 (51.0%), NNRTI in 84/382 (22.0%) and INSTI in 67/382 (17.5%) patients. Other unconventional regimens were used in a small subset of patients: PI/r-based combinations in 16/382 (4.2%) or other combinations in 20/382 (5.2%) patients. The reason for switching was simplification for 256/382 (67.0%) patients, tolerance issues for 85/382 (22.0%) and various other reasons for 41/382 (10.8%) of the patients.

### Efficacy results (Table 2)

**Table 2:** primary endpoint analysis at W48 of the 382 patients switching to E/C/F/TDF

	Present study n = 382	S trategy–PI Arribas et al. [9] n = 290	Strategy– NNRTI Pozniak et al. [10] n = 290
Success (patients with VL < 50 copies/mL at W48)	314 (82.0) [95% CI 78.4–86.0]	272 (94.0)	271 (93.0)
Virologic failure (VL ≥ 50 copies/mL at W48)	13(3.5%) [95% CI 3.64–8.41]	2 (1.0)	3 (1.0)
Genotype available at failure	6/13	0 (0.0)	1
Resistance to INSTIs	5/6	0 (0.0)	0
No data	55 (14.5) 95% CI 10.9–17.9]	16 (6.0)	16 (6.0)
Adverse events leading to treatment discontinuation	23 (6.0) [95% CI 3.6–8.4]	6 (2.0)	6 (2.0)
Other reasons for treatment discontinuation and lost to follow–up	31 (8.2)* [95% CI 5.6–11.4]	11 (3.8)	11 (3.8)
Death	1 (0.3) [95% CI 0.0–1.7]	0 (0.0)	1 (0.3)
Total	382 (100.0)	290 (100.0)	290 (100.0)

Data are n (%) [95% CI]

*INSTI* integrase strand transfer inhibitor

*Lost to follow-up (n = 16), patient’s decision (n = 4), drug-drug interaction (n = 3), pregnancy (n = 3), unknown reason (n = 3), toxicity prevention (n = 1), end of treatment (n = 1)

At W48, using the FDA snapshot analysis, 314 (82.0% [95%CI 78.4–86.0]) patients met the definition of success with HIV RNA < 50 copies/mL while still on E/C/F/TDF and 13 (3.5% [95%CI 3.64–8.41]) experienced virologic failure, 23 (6.0% [95%CI 3.6–8.4]) discontinued E/C/F/TDF due to an adverse event, 31 (8.2% [95%CI 5.6–11.4]) discontinued treatment for other reasons or were lost to follow-up and 1 patient died (myocardial infarction).

### Virologic failure and resistance (Table 3)

**Table 3:** Description of patients with virologic failure

Patients with virologic failure n=13	Previous ART (just before switching)	Previous ART duration	VL at failure (copies/mL)	VL confirmation	Time of VF after the switch	Previous VF^§^	Previous INSTI exposure	Previous genotypic test		Genotypic test at VF	
								NRTI RAMs	INSTI RAMs	NRTI RAMs	INSTI RAMs
VL>50 copies/mL between W0 and W48 leading to treatment discontinuation											
Patient 01	TDF/FTC+ ATV/r	3.7 years	10257	418	1.5 months	Yes	Yes	L74V, M184V	N155H	L74V, M184V	N155H
Patient 02	TDF/FTC+DRV/r	3 years	65679	110664	5 months	No	No	T215N/Y/S, M184V	ND	L74V, M184V	N155H
Patient 03	TDF/FTC+DRV/r	3 years	77	180370	3 months	No	No	T215A/C/D/E/G/H T215I/L/N/S/V	ND	41L,M184V,T215Y	L74F/I,Q148H/R/K
Patient 04	TDF/FTC+DRV/r	7 months	37791	16041	2 months	Yes	Yes	None	None	NA	NA
VL>50 copies/mL between W0 and W48 without treatment discontinuation (or LTFU)											
Patient 05*	TDF/FTC+ ATV/r	8 months	2918	ND	4 months	NA	No	M184V	ND	ND	ND
Patient 06*	TDF/FTC+DRV/r	1 year	60	112	2 months	NA	No	ND	ND	ND	ND
Patient 07*	TDF/FTC+DRV/r	2.2 years	112	ND	5 months	NA	No	NA	NA	ND	ND
Patient 08**	TDF/FTC+RAL	4 years	91	158	6 months	Yes	Yes	NA	NA	ND	ND
Vl>50 copies/mL at W48											
Patient 09	TDF/FTC+RAL	2.3 years	453	149	11 months	No	Yes	None	None	No amplification	No amplification
Patient 10	TDF/FTC+FPV/r	7 years	6830	4990	12 months	Yes	No	D67N, K70R,L74V,M184V/I,T215Y/F, K219Q/E	ND	D67N, K70R,M184V/I,S215F, K219Q/E	N155H
Patient 11	ABC/3TC+ATV	9 years	169	594	12 months	NA	No	NA	NA	D67N, K70R,M184V/I,K219Q/E	N155H
Patient 12	TDF/FTC+ ATV/r	9 months	3339	22957	12 months	Yes	No	None	None	None	None
Patient 13	TDF/FTC/EFV	2.5 years	1767	ND	12 months	Yes	No	None	ND	ND	ND

*ART* antiretroviral therapy, *INSTI* integrase strand inhibitor, *NRTI* nucleoside reverse transcriptase inhibitor, *RAM* resistance associated mutations, *VL* viral load, *VF* virologic failure; *NA* information not available in the data base, *ND* not done, *LTFU* lost to follow-up

*TDF/FTC* tenofovir/emtricitabine, *EFV* efavirenz, *DRV/r* darunavir boosted with ritonavir, *ATV* atazanavir, *ATV/r* atazanavir boosted with ritonavir, *RAL* raltegravir, *FPV/r* fosamprenavir boosted with ritonavir

^§^Previous VF based on the mention in the patient’s therapeutic history that ART was changed because of VF

*These patients were lost to follow-up after the diagnosis of VF but patient 7 never stopped treatment and returned to his center in sept 2017 with undetectable HIV RNA while still on E/C/F/TDF

**This patient continued E/C/F/TDF for an additional year but with persistent low levels viraemia. 18 months later, INSTI associated resistance was confirmed and the patient switched to PI/r

Thirteen (3.5%) patients experienced virologic failure: eight had an HIV RNA ≥ 50 copies before W48 and five at week 48.

Results of genotypic resistance at the time of virologic failure were available for six patients and not performed or not available for the seven remaining patients. Among the six patients with available genotype, five had NRTI and INSTI RAMs. Out of these five patients, comparison with genotype prior to the switch was possible in four: all had NRTI RAMs and one had INSTI RAMs.

### Risk factors for virologic failure (Table 4)

**Table 4:** Risk factors for virologic failure

	Virological failure n = 13, n/median (%/IQR)	Virological success n = 314, n/median (%/IQR)	p–value
Age, years	46 (41–50)	44 (38–51)	0.82
Gender, male	10 (77.0%)	219 (70.0%)	0.76
Mode of HIV infection	–	–	1.00
Heterosexual	7 (54.0%)	162 (52.0%)	–
MSM	5 (38.0%)	117 (37.0%)	–
Others	1 (8.0%)	35 (11.0%)	–
Nadir CD4 cell count (cell/mm ^3^ )	212 [166–255]	231 [107–349]	0.77
CD4 cell count at baseline (cell/mm ^3^ )	536 [405–866]	619 [452–804]	0.89
Creatinine levels at baseline (µmoL/L)	79 [68–88]	79 [70–90]	0.76
Time since HIV infection, years	12 (11–19)	11 (5–17)	0.27
Duration of HIV treatment, years	9 (6–10)	8 (4–13)	0.77
Number of previous ART regimens	4 (2–4)	3 (2–5)	> 0.99
History of VF before the switch	2 (15.0%)	64 (20.0%)	1.00
Duration of HIV RNA < 50 copies/mL before the switch, months	21.9 (3–26)	33.6 (13–63)	0.05
INSTI base ART at time of switch	2 (15.0%)	66 (21.0%)	1.00
PI/r based ARTat time of switch	9 (69.2%)	177 (56.4%)	0.44
Previous M84V/I*	4/9 (44.0%)	27/143 (19.0%)	0.09
Previous M84V/I + at least 1 other TDF RAM* (among M41L,E44D, D67N,T69D/N/S, L74V/I, L210W or T215A/C/D/E/G/H//L/N/S/V/Y/F)	3/9 (33.3%)	14/143 (9.8%)	0.06
Previous K65R*	0/9 (0.0%)	1/143 (1.0%)	1.00
Previous RAMs to elvitegravir (N155H)*^§^	1/9 (11.0%)	0/143 (0.0%)	0.06

*RAM* resistance associated mutations, *MSM* men who have sex with men, *VF* virologic failure, *INSTI* integrase strand transfernhibitors, *TDF* tenofovir, *3TC* lamivudine, *FTC* emtricitabine

§this patient had also M184V

*For the 314 patients with virologic success, 143 had a genotype available at baseline in the electronic health record (EHR); for the 13 patients with virologic failure, genotypes were available for 9, either available in the EHR or retrospectively collected at the virology lab

A genotypic test for resistance was available at baseline for nine of the 13 patients with virologic failure (69.0%) and 143 of the 314 patients with virologic success (46.0%).

There was no significant difference between the two groups in terms of baseline characteristics or presence of resistance-associated mutations to NRTIs except that patients who experienced failure had undetectable viremia for a shorter period (21.9 vs. 33.6 months, p = 0.05). Among the patients who failed, M184V and other NRTI RAM conferring resistance to TDF were more frequent, but the difference was not statistically significant.

### Safety results (data not shown)

There was a significant change in creatinine levels between the switch and W48 but not clinically meaningful: the median creatinine level was 79 µmol/L (IQR 70–89) at baseline and 85 (IQR 75–96) at W48 (p < 0.001); data not shown; among the 382 patients, 38 and 57 creatinine values were missing at baseline and W48 respectively.

All patients who discontinued E/C/F/TDF between the switch and W48 were analyzed to describe reasons for treatment discontinuation and identify clinically significant adverse events leading to treatment discontinuation. Among the 43 treatment discontinuations, 23/43 (54.0%) were related to mild adverse events: 10 gastro-intestinal, three renal adverse events and two mild transaminase elevation; other various adverse events were seen in eight patients (nightmares in two, fatigue or muscular pain in two, tachycardia in one and reason was not specified for three patients). The three renal adverse events were laboratory abnormalities and patients were asymptomatic: one hypophosphatemia and two creatinine level elevation of less than 20%.

## Discussion

In our retrospective cohort study of PWH fully suppressed on ART, the proportion of patients maintaining a plasma HIV RNA level < 50 copies/mL one year after switching to E/C/F/TDF was 82.0%. This proportion remains however lower than that reported in randomized clinical trials where success rates above 93% have been reported with a switch to E/C/F/TDF or E/C/F/TAF [8, 9, 18–20]. These differences can be explained in part by a higher rate of patients discontinuing the study drugs because of drug-related adverse events, lost to follow-up or other reasons in our study (14.0% ) as compared to prior randomized trials (4–6%) [8, 9, 19]. Also, contrary to prior randomized studies, we allowed in our study patients with prior virologic failure (24.6%), those with more than two different ART regimens before the switch (75.0%) and those with prior integrase inhibitors-based ART regimens (17.5%). Our study has therefore enrolled patients that are more representative of real-life practice.

These differences can also explain the higher rate of virologic failure in our study (3.5%) as compared to prior studies (1.0%) (Table 2) [8, 9, 18, 20]. An additional reason for this higher rate of virologic non response is that we allowed patients with prior NRTI or integrase resistance mutations to be enrolled, whereas these patients were excluded from the analysis in prior randomized trials [8, 9]. Indeed, in our study among 152 patients with an HIV resistance genotype available before the switch, 31/152 (20.3%), and 17/152 (11.2%) had the M184V/I and M184V/I + at least one TDF-associated resistance mutation identified, respectively (Table 3).

When we assessed baseline risk factors associated with virologic failure, we could not identify prior resistance associated mutations as a significant risk factor, possibly because of the low number of patients with virologic failure. However, previous cohort studies have also shown, that pre-existing NRTI resistance associated mutations were not associated with the risk of emerging resistance [21–25]. Among 15 patients switching to E/C/F/TDF with previous mutations to FTC/TDF, only one experienced virological failure [23]. In two studies enrolling patients with M184V and receiving a TDF or abacavir-based regimen switching to elvitegravir based ART, there was no impact of the presence of M184V on virologic success [22, 24].

The only factor we found associated with virologic failure was the duration of viral suppression before the switch which was 21.9 months in those with virologic failure as compared to 33.6 months in those without virologic failure (p = 0.05). This association between the duration of viral suppression and a lower risk of virologic failure is consistent with prior studies [26, 27].

Of interest, and contrary to randomized trials and cohort studies, we report a high rate of integrase inhibitor-associated mutations in patients with virologic failure with 5/6 (83.3%) patients showing at the time of failure the N155H (n = 4) or Q148H (n = 1) mutations (Table 3). All five patients also had NRTI-associated resistance mutations which were already detected in genotypes before the switch in four out of five patients. This contrasts with previous reports where integrase inhibitor resistance was either not found at the time of failure or identified in a single patient in one study [8, 9, 18–20]. Our results are consistent with a low genetic barrier to resistance with elvitegravir as compared to second- generation integrase inhibitors. There are no trials comparing face-to-face elvitegravir to either dolutegravir or bictegravir. However, the results of a previous study comparing elvitegravir/cobicistat to raltegravir in patients naïve to integrase inhibitor but failing current ART showed a similar virologic failure rate with both regimens and a similar rate of emergence of resistance to integrase inhibitors (27% and 21%, with elvitegravir and raltegravir, respectively) [28]. In addition, in a similar population, dolutegravir outperformed raltegravir with a lower rate of virologic failure, and fewer patients developed integrase resistance in case of virologic failure with dolutegravir as compared to raltegravir (4/45 (8.9%) vs. 17/21 (80.9%), respectively [29]. These results explain in part why elvitegravir is no longer recommended as a preferred integrase inhibitor in most guidelines [13, 14].

In our patients, the safety and tolerability of E/C/F/TDF were good. The rate of study drug discontinuation, remained low (6.0%) although higher than in prior randomized studies and was due to mild adverse events or laboratory abnormalities, such as gastro-intestinal-related adverse events.

Our study has however several of limitations. Due to its retrospective design, a number of data are missing, especially regarding prior genotypic resistance tests. The low number of patients with virologic failure also precluded a more detailed analysis of risk factors associated with this outcome. Follow-up was short and the rate of study discontinuation was higher than in prospective studies.

The strength of our study is to have enrolled patients with different treatment histories, and to provide an opportunity to better assess the antiviral activity and genetic barrier to resistance of E/C/F/TDF in these difficult-to-treat patients in a real-world setting. Indeed, our results underline the importance of analyzing in detailed treatment history when considering a switch to elvitegravir in well-suppressed patients. Caution is required with close monitoring of plasma HIV-1 RNA levels, if patients have experienced previous treatment failure, have prior NRTI or integrase resistance mutations or no resistance genotype available or in case of a short duration of viral suppression. In these cases, switching to a drug with a higher genetic barrier to resistance is advisable.

## Data Availability

Data will not be shared publicly. Data could be made available to any researcher interested. Deidentified participant data would be made available with a data dictionary and shared under a Data Transfer Agreement. Data can be requested at the following email address: nathalie.de-castro@aphp.fr.
